# Allosteric Differentiation of Al(I) Reactivity

**DOI:** 10.1002/chem.202501352

**Published:** 2025-04-25

**Authors:** Han‐Ying Liu, Jakub Kenar, Henry T. W. Shere, Ryan J. Schwamm, Michael S. Hill, Mary F. Mahon, Claire L. McMullin

**Affiliations:** ^1^ Department of Chemistry University of Bath, Claverton Down Bath BA2 7AY UK

**Keywords:** alkyne, aluminum, arene activation, cycloaddition, density functional theory

## Abstract

The dimeric potassium alumanyl, [{SiN^Dipp^}AlK]_2_ ({SiN^Dipp^} = {CH_2_SiMe_2_N(Dipp)}_2_; Dipp = 2,6‐*i*‐Pr_2_C_6_H_3_), reacts with two equivalents of PhC≡CR (R = Ph or SiMe_3_) with the exclusive formation of the aluminacyclopropene derivatives, [{SiN^Dipp^}Al{η^2^‐C_2_(R^1^)(R^2^)}]. In contrast, reactions with an equal stoichiometry of both alumanyl and alkyne reagents provide the products of not only alkyne cycloaddition but also *para*‐C–H activation of a phenyl substituent. Supported by a theoretical study, this outcome is judged to result from a sequence of cooperative steps and the introduction of a modicum of kinetic discrimination that is suggested to be allosteric in origin.

## Graphical abstract

A dimeric potassium alumanyl reacts with phenyl‐substituted alkynes to provide not only alkyne cycloaddition but also *para*‐C–H activation of the phenyl substituent. Supported by a theoretical study, this outcome is judged to arise from a sequence of cooperative steps and a mode of kinetic discrimination that is suggested to be allosteric in origin.

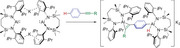

## 1 Introduction

Allosteric effects, whereby binding of an “effector” substrate prompts a conformational change at a proximal or distal region so as to activate a secondary reactive site, are widely recognized to mediate cellular metabolism and the function of many proteins and enzymes.^[^ ^1^ ^]^ The design of synthetic organic structures that similarly respond to an applied stimulus dates from the work of Rebek Jr. in the late 1970s.^[^ ^2^ ^]^ The concept of allosteric regulation has since inspired significant progress in the development of many supramolecular systems, particularly those in which a conformational and/or dynamic change is induced through the cooperative binding of a cation and in which a metal center itself plays the role of effector.^[^ ^3^ ^]^ Conformational unveiling of a reactive transition metal site to instigate a variety of catalytic, energy transfer, or redox‐based processes has also been achieved through the deliberate introduction of “weak links” (e.g., π–π stacked interactions) within more complex metal–organic structures.^[^ ^4^ ^]^ While the electronic consequences of the binding of a molecular effector, for example, an inorganic Lewis acid, at a location remote from the active site and the modulation of transition metal reactivity have also been recognized,^[^ ^5^ ^]^ to the best of our knowledge, the potential for allosteric effects has not been identified in any instance of *p*‐block or main group element‐centered reactivity. In this contribution, therefore, we demonstrate that the robust but labile links provided by the potassium‐arene interactions of a dimeric Al(I) (alumanyl) anion may be purposely exploited to effect kinetically discriminated dual C≡C and Ar(C–H) activation of phenyl‐substituted alkynes.

**Figure 1:**
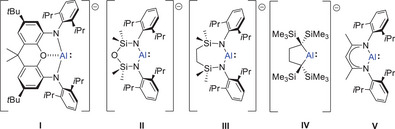
The *N*,*N*′‐(diamido)alumanyl anions, **I**–**III**, the *C,C*′‐(dialkyl)alumanyl anion, **IV**, and the structure of compound **V**.

Pioneered by the groups of Aldridge, Goicoechea, and Coles, the chemistry of alumanyl anions (e.g., **I** and **II**, Figure 1) has emerged as a fertile area of contemporary study.^[^ ^6^ ^]^ Despite dating from only 2018, a potent combination of highly reducing character and Al‐centered nucleophilicity has delivered a panoply of striking small molecule activation processes^[^ ^7^, ^8^, ^9^, ^10^ ^]^ and unprecedented aluminum‐to‐heterometal σ bonds.^[^ ^11^ ^]^ Our own modest contribution to this area has centered on the development of the chemistry of the seven‐membered cyclic diamidoalumanyl anion, [{SiN^Dipp^}Al]^−^ (**III**, {SiN^Dipp^} = {CH_2_SiMe_2_N(Dipp)}_2_; Dipp = 2,6‐*i*‐Pr_2_C_6_H_3_), which displays similar attributes as a soluble two‐electron reductant and as a Group 13 nucleophile.^[^ ^12^ ^]^

**Figure 2:**
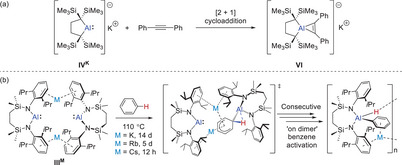
(a) The [2 + 1] cycloaddition of diphenylacetylene induced at **IV^K^**; (b) the C–H activation of benzene by dimeric **III^M^** and the gradation of reactivity resulting from variation of M (M = K, Rb, Cs).

Characteristic alumanyl reactivity is provided by a propensity toward [2 + 1] cycloaddition with molecules containing C–C unsaturation. The groups of Aldridge and Coles have, for example, reported that the potassium derivatives of **I** and **II** (henceforth denoted as **I^K^** and **II^K^**) react with ethene under mild conditions (1 bar, room temperature) to provide the corresponding aluminacyclopropanes.^[^ ^6^, ^7^, ^9^ ^]^ Roesky and co‐workers demonstrated that treatment of the charge‐neutral β‐diketiminato (BDI) Al(I) species **V** (Figure 1) with ethyne or mono‐ and di‐substituted alkynes provides the corresponding aluminacyclopropenes, [(BDI)Al{η^2^‐C_2_(R^1^)(R^2^)}] (e.g., R^1^ = R^2^ = H; R^1^ = H, R^2^ = Ph; R^1^ = R^2^ = Me),^[^ ^13^, ^14^ ^]^ with theoretical studies indicating that twofold Al─C bond formation is effectively barrierless but occurs asynchronously.^[^ ^14d^ ^]^ Among alumanyl species, Yamashita has shown that analogous diphenylacetylene addition may be induced at the dialkyl variant **IV^K^** to provide the aluminate analogue (**VI**, Figure 2a).^[^ ^9b^ ^]^ In contrast, we have recently observed that reactions of terminal alkynes with the [{SiN^Dipp^}Al]^−^ anion result in oxidative addition of the acidic C─H bonds and the generation of hydrido(alkynyl)aluminates, [{SiN^Dipp^}Al(H)(C≡CR)]^−^.^[^ ^15^ ^]^ The chemistry of these and other formally anionic species is also marked by an ability to effect the oxidative addition of the kinetically robust *sp* ^2^‐C─H bonds of arene derivatives (e.g., for **III^M^** where M = Na, K, Rb, or Cs, Figure 2b).^[^ ^6^, ^7^, ^9^, ^16^ ^]^ Although generally requiring higher temperatures, it is notable that similar reactivity is only precedented in the chemistry of **IV** under Pd‐catalyzed conditions.^[^ ^17^ ^]^

The anionic structures depicted in Figure 1 neglect to portray that the anions **I**–**IV** are necessarily combined with an alkali metal cation (M^+^). While charge balance is maintained in a majority of reported cases by potassium (**I^K^** ^–^ **IV^K^**), examples have also now been described for the complete range of available Group 1 metals.^[^ ^8^, ^11^, ^16^, ^18^ ^]^ Irrespective of Group 1 metal identity, a characteristic solid‐state feature resulting from the combination of the *N‐*aryl derivatives **I^M^**–**III^M^** with the heavier members of this series (i.e., M = K, Rb, Cs) is the adoption of a dimeric contact ion pair structure, in which two alumanyl moieties are connected by multihapto M^+^∙∙∙(arene) interactions via the flanking *N*‐aryl substituents. While the identity and size of the M^+^ cations exert some structural influence,^[^ ^6d^ ^]^ more potential significance arises from the impact of M^+^ identity on the kinetic viability of the reactivity exemplified in Figure 2b. Computational (DFT) assessment of benzene C–H activation by **III^K/Rb/Cs^** has indicated that rate‐determining attack of the Al(I) nucleophile is a cooperative process during which the dimeric structure is retained but is increasingly enabled by π‐engagement of the arene with the M^+^ cation as Group 1 is descended.^[^ ^16^ ^]^ Calculations on the reactivity of **III^K/Rb/Cs^** with the C─H bonds of terminal alkynes inferred that oxidative addition at Al also proceeds with retention of the initial dimeric alumanyl structure.^[^ ^15^ ^]^ In this case, however, kinetic differentiation across the three alkali metals was deduced to reflect the relative stability of the [{SiN^Dipp^}AlM]_2_ dimers (K > Rb > Cs).

These observations prompted us to consider whether the persistent dimeric structure of **III^K^** could be exploited to elicit two kinetically and chemically discriminated reaction steps in a single molecular transformation. In this contribution we show that the kinetic facility of [2 + 1] PhC≡CR (R = Ph; SiMe_3_) cycloaddition and *para*‐C–H activation of the subsequently appended aryl substituent permits their consecutive reaction at the Al(I) centers of the dimeric alumanyl, [{SiN^Dipp^}AlK] (**III^K^**), providing a unique means to effect the twofold activation of phenyl‐substituted internal alkynes.

## 2 Results and Discussion

**Scheme 1:**
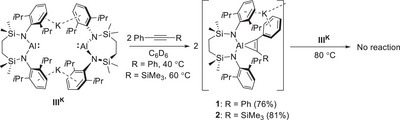
Synthesis of compounds **1** and **2** and their subsequent lack of reactivity toward **III^K^**.

**Figure 3:**
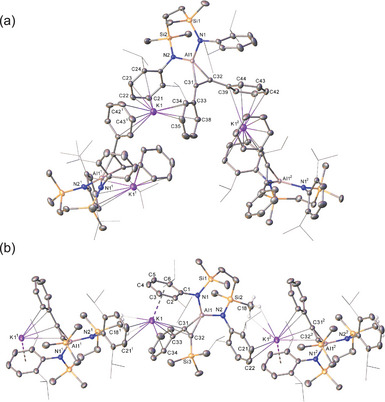
(a) Plot depicting the molecular structure of **1**. Ellipsoids are depicted at 30% probability. Hydrogen atoms have been omitted and peripheral substituents are depicted as wireframes, for clarity. Symmetry operations: ^1^ 1 + *y* − *x*, 2 − *x*, ^1^∕_3_ + *z*; ^2^ 2 − *y*, 1 + *x* − *y*, − ^1^∕_3_ + *z*. (b) Plot depicting the structure of **2**. Ellipsoids are depicted at 30% probability. Minor disordered components have been omitted as have hydrogen atoms (those attached to C18 excepted), for clarity. Peripheral substituents are depicted as wireframes, also for visual ease. Symmetry operations: ^1 3^∕2 − *x*, − ½ + *y*, − ½ + *z*; ^2 3^∕_2_ − *x*, ½ + *y*, ½ + *z*.

To confirm the viability of the mooted [2 + 1] cycloaddition, initial experiments were performed in a 2:1 stoichiometry between both diphenylacetylene and trimethyl(2‐phenylethynyl)silane and **III^K^** (Scheme 1). Although the reactions required heating at 40°C and 60°C, respectively, to achieve quantitative conversion, good yields of the crystalline aluminacyclopropene derivatives, compounds **1** (76%) and **2** (81%), were isolated from toluene or benzene solutions. While a C_6_D_6_ solution of the yellow η^2^‐*C*,*C*′‐diphenyl‐substituted species **1** provided a ^1^H NMR spectrum consistent with *C*_2_ symmetry, the corresponding {SiN^Dipp^} ligand environments of the dark red silyl derivative, **2**, while assignable, were broadened and indicative of lower local symmetry about the aluminum center. This inference was confirmed by single crystal X‐ray diffraction analysis of both compounds, the results of which are shown in Figure 3. Although the asymmetric units of both **1** and **2** constitute a monomer, their structures are dominated by 1‐dimensional polymers, which propagate about the screw axes intrinsic to their respective crystallographic space groups. The Al1–C31–C32 metallocycles form *pseudo*‐isosceles triangles with Al─C bond lengths [**1**: Al1–C31 1.955(3), Al1–C32 1.928(2); **2**: 1.932(2), 1.942(2) Å] that are somewhat elongated in comparison to, for example, Roesky's aluminacyclopropene, [HC{(Me)CNDipp}_2_Al(η^2^‐C_2_Ph_2_)] [1899(3), 1.908(3) Å],^[^ ^14c^ ^]^ but are commensurate with the corresponding bonds within the aluminate analogue **VI** [Figure 2a, 1.945(3), 1.940(3) Å].^[^ ^9b^ ^]^ While this discrepancy likely reflects the impact of the presence of potassium in both **1**, **2,** and **VI**, the alkali metal cation interacts symmetrically with both phenyl substituents of the aluminacyclopropenyl unit in the previously described derivative. In contrast, the alkali metal centers of both **1** and **2** engage with a single aluminacyclopropene phenyl substituent of each [{SiN^Dipp^}Al(C_2_PhR)]^−^ moiety and via polyhapto intra‐ and intermolecular interactions with the *N‐*aryl groups of each {SiN^Dipp^} supporting ligand.

**Scheme 2:**
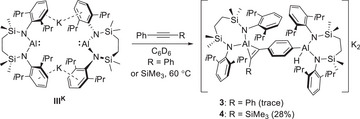
Synthesis of compounds **3** and **4**.

**Figure 4:**
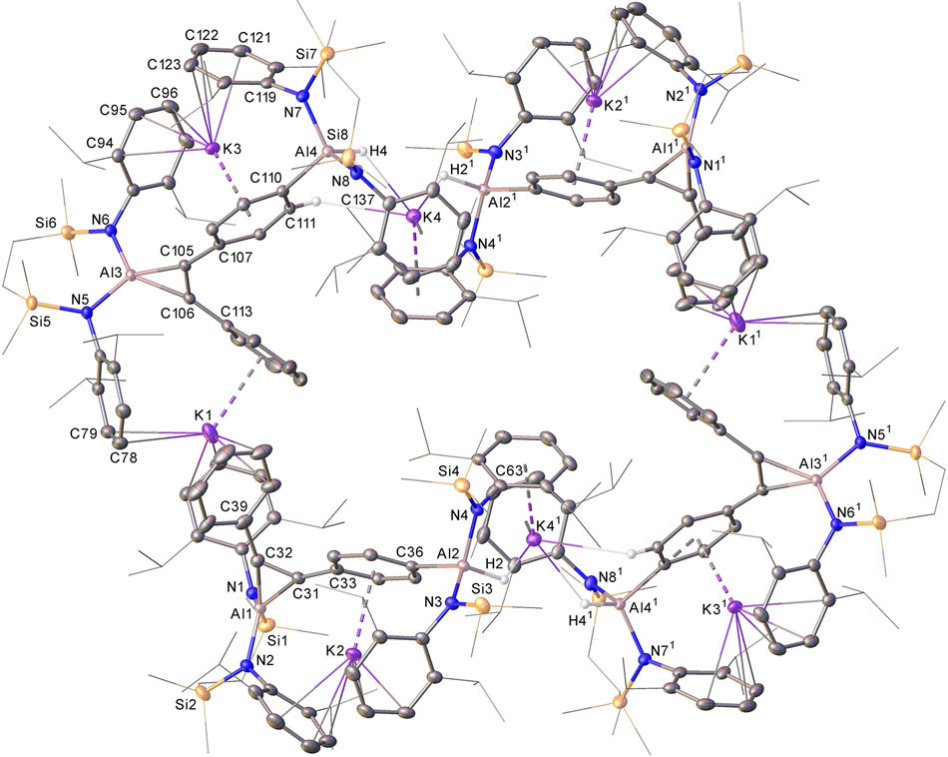
Plot depicting the structure of **3**. Ellipsoids depicted at 30% probability. Solvent, minor disordered components and hydrogen atoms (H1, H2, H3, H4, and H111 excepted) omitted for perspicuity. Peripheral substituents are depicted as wireframes, also for clarity. Symmetry operations: ^1^ 1 − *x*, 1 − *y*, − *z*.

With viable quantities of compounds **1** and **2** in hand, their reactivity toward a further equivalent of **III^K^** was assessed in C_6_D_6_. Although both reactions were heated to 80°C, a temperature at which solvent activation becomes apparent,^[^ ^16b^ ^]^ no evidence of any reaction with either aluminocyclopropene derivative could be identified (Scheme 1). Further studies, therefore, focused on the reactivity of a single equivalent of each internal alkyne per alumanyl dimer. Monitoring of C_6_D_6_ solutions of [{SiN^Dipp^}AlK]_2_ with PhC≡CPh heated either at 80°C or 60°C by ^1^H NMR spectroscopy continued to imply the predominant formation of compound **1**. The latter reaction, however, was accompanied by the generation of a further insoluble product (**3**), which deposited directly from the reaction solution as a small quantity of orange needle crystals (Scheme 2). Although attempts to isolate a bulk sample of **3** for further solution characterization and microanalysis were unsuccessful, it was identified by X‐ray diffraction analysis (Figure 4). The structure of **3** is the product of not only [2 + 1] alkyne cycloaddition at aluminum but also C–H oxidative addition at a second [{SiN^Dipp^}Al] unit of a *para*‐situated *C*‐phenyl methine of the resultant aluminacyclopropene moiety.

The asymmetric unit of **3** contains four aluminum and potassium centers and further dimerizes by way of crystallographic inversion symmetry to form cyclical arrays. Propagation of the octameric structure is, thus, achieved by a network of polyhapto K∙∙∙Ph and K∙∙∙Dipp interactions. Whereas K1, K2, and K3 are entirely encapsulated by both {SiN^Dipp^} ligands and the phenyl substituents of the cyclopropenylaluminate anions bound to Al1 and Al3, the binding of K4 (K4') is augmented by Al–H coordination provided by Al2 (Al2') and Al4 (Al4'). The coordination environments of the distorted tetrahedral Al2 and Al4 centers are each completed by covalent bonds to the *para*‐carbon atoms of one of the cyclopropenylaluminate aryl substituents, while the other is retained as an unperturbed phenyl group.

**Figure 5:**
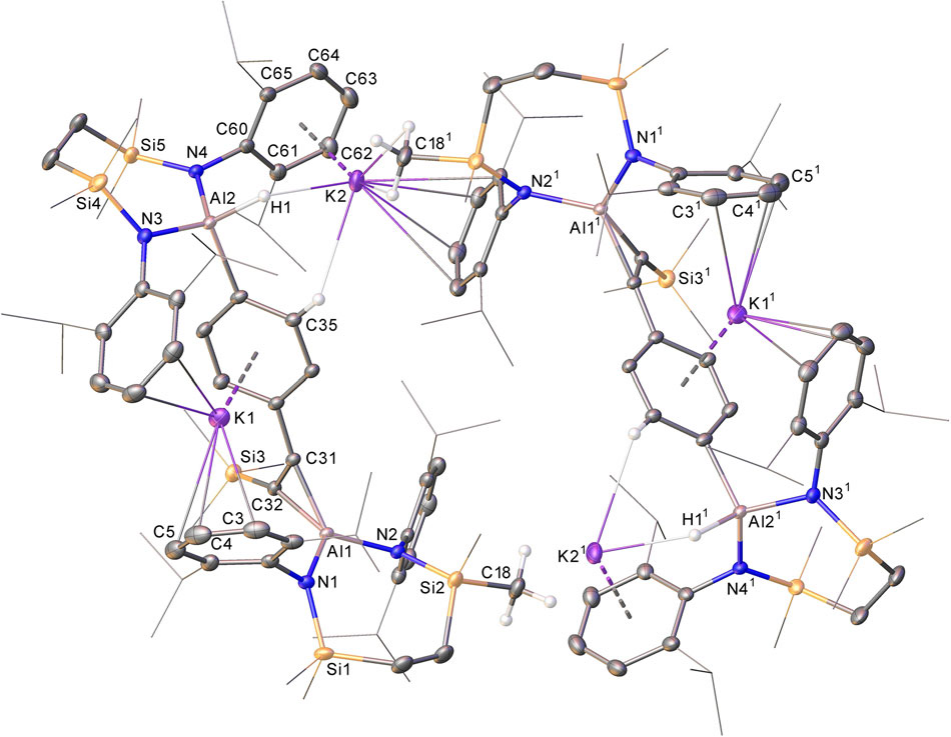
Plot depicting the structure of **4**. Ellipsoids are depicted at 30% probability. Hydrogen atoms (H1, H2 and those attached to C18 and C35 excepted) have been omitted and peripheral substituents are depicted as wireframes, for clarity. Symmetry operations: ^1^ 1 − *x*, ½ + *y*, 1 − *z*.

A reaction performed at 80°C with an equal stoichiometry of the **III^K^** dimer and PhC≡CSiMe_3_ also provided consumption of only 50% of the alumanyl reagent and effectively exclusive formation of **2**. An otherwise identical reaction heated at 60°C for one week in C_6_D_6_, however, led to the deposition of a dark red crystalline compound (**4**) that could be isolated in 28% yield and was identified by X‐ray diffraction analysis as a further product of twofold alkyne activation analogous to that leading to compound **3** (Figure 5). The structure shares many common features with **3**, albeit its asymmetric unit comprises a single dipotassium aluminate ion pair, [{SiN^Dipp^}_2_Al(H)(4‐C_6_H_4_C_2_(SiMe_3_)‐η^2^‐Al{SiN^Dipp^}]K_2_. Although these individual moieties propagate as puckered 1‐dimensional polymers parallel to the crystallographic *b*‐axis, compound **4** redissolved readily in d*_8_*‐THF. The resultant analysis by NMR spectroscopy provided data that supported the retention of the dialuminated alkyne substrate in solution. Resumably as a consequence of the quadrupolar (*I* = ^5^/_2_) ^27^Al centers, neither the alumuninacyclopropene ^13^C environments nor the Al‐bound phenyl carbon nucleus could be observed. In contrast, the 2,6‐ and 3,5‐methine C–H environments arising from the formation of the 4‐*C*─Al bond could be discriminated as two well‐resolved doublet resonances (*δ*_H_ 7.31, 6.61 ppm; ^3^ *J* = 7.2 Hz) in the ^1^H NMR spectrum, while the corresponding ^13^C environments (*δ*_C_ 137.4, 121.0 ppm) were readily identified by an HSQC experiment.

**Figure 6:**
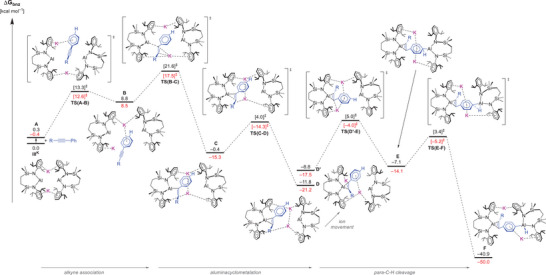
Computed free energy profile (BP86‐D3^BJ^(PCM = C_6_H_6_)/BS2//BP86/BS1 level, energies quoted in kcal mol^−1^) for the formation of the dialuminated alkyne product, **F** (**3** and **4**), through initial aluminacyclometalation, followed by *para* C–H cleavage of Ph. Free energies when R = Ph are in red; free energies when R = SiMe_3_ are in black.

The processes leading to compounds **3** and **4** (hereafter denoted as **^Ph^F** and **^SiMe3^F**), were assessed by density functional theory (DFT). Mechanisms instigated by complete cleavage of the dimeric potassium alumanyl were investigated alongside pathways in which the dialumanyl constitution of **III^K^** is retained. In the former cases, and consistent with previous observations of benzene activation induced by a monomeric form of **III^K^**,^[^ ^16b^ ^]^ the barrier heights associated with the aluminacyclopropanation process were computed to be of the order *ca*. 40 kcal mol^−1^ (see Figure S11 and the Supporting Information for full details). The preferred mode of reaction, thus, invokes dissociation of a single *N*‐Dipp substituent from potassium and a resultant “hinging” of the alumanyl dimer, which pivots at the remaining alkali metal cation (Figure 6) to allow access to the alkyne substrate. Although an alternative reaction profile retaining the dimeric structure but with initial *para*‐activation of the alkyne phenyl substituent was also identified (see ESI), from this point the more kinetically viable trajectory first leads to the assembly of an aluminacyclopropene intermediate (**^Ph^D** = −21.2; **^SiMe3^D** = −11.8 kcal mol^−1^) at a single Al(I) center.

Consistent with Roesky and co‐workers’ earlier calculations,^[^ ^14d^ ^]^ the two Al─C bonds are formed asynchronously via a κ^1^ intermediate (**^Ph^C** = −15.3, **^SiMe3^C** = −0.4 kcal mol^−1^), which positions both potassium cations between the alkyne and the second Al(I) center. The alkyne κ^1^ coordination transition state, **TS(B‐C)**, provides the highest barrier at 17.5 and 21.6 kcal mol^−1^ for R = Ph and SiMe_3_, respectively. Despite its apparent facility, this process is found to be rate‐limiting, whereupon the transformation of **D** to **F** ensues through a two‐step process initiated by the assembly of a Meisenheimer intermediate (**E**) resulting from the attack of the now remaining Al(I) center. While this process represents the more energetically demanding step of the overall C–H addition process (Δ*G*_bnz_^‡^; **^Ph^TS(D′‐E)** = 13.5, **^SiMe3^TS(D′‐E)** = 13.8 kcal mol^−1^), the anchimeric assistance provided by engagement of both K^+^ ions with the aryl π system and the now proximal orientation of the Al(I) center ensures that reductive cleavage of the C–H (Δ*G*_bnz_^‡^; **^Ph^TS(E‐F)** = 8.9, **^SiMe3^TS(E‐F)** = 10.5 kcal mol^−1^) occurs exergonically to form **F** (**^Ph^F** = −50.0 / **^SiMe3^F** = −40.9 kcal mol^−1^).

## 3 Conclusions

In conclusion, this work demonstrates that two points of activation of a single organic small molecule may be achieved at a dimeric Al(I) reagent. We suggest that this behavior may be attributed to the persistent yet labile interactions between the potassium cations and the flanking *N*‐aryl substituents of the supporting diamide ligand. Viewed from this perspective, the environment provided by the {SiN^Dipp^} dianions impacts not only the stability of the Al(I) centers but also their independent operation. The alkali metal cations can be seen to also exert a secondary role in their response to the conformational change induced by the initial reaction step. This impacts significantly on the barrier height associated with the otherwise more energetically demanding process of aryl activation. These observations may, thus, be identified as allosteric in origin, with the alkyne reagent adopting the role of effector. We are continuing to assess the potential of this behavior and its application to alternative small molecule substrates and in other reactive main group element systems.

## Supporting Information

Full experimental and instrumental details, NMR spectra, details of the x‐ray analysis and the methods employed in the quantum chemical investigations of this chemistry are available in the Supporting Information to this article, including additional computational discussion. Crystallographic data for all compounds have been deposited with the Cambridge Crystallographic Data Centre as supplementary publications CCDC 2,366,296–2,366,299 for **1**–**4**, respectively. Copies of these data can be obtained free of charge on application to CCDC, 12 Union Road, Cambridge CB2 1EZ, UK [fax(+44) 1223 336033, e‐mail: deposit@ccdc.cam.ac.uk.

## Conflict of Interests

The authors declare no conflicts of interest.

## Supporting information



Supporting Information

## Data Availability

The data that support the findings of this study are available in the supporting information of this article.
